# Environmental impact of the largest petroleum terminal in SE Brazil: A multiproxy analysis based on sediment geochemistry and living benthic foraminifera

**DOI:** 10.1371/journal.pone.0191446

**Published:** 2018-02-12

**Authors:** Wânia Duleba, Andreia C. Teodoro, Jean-Pierre Debenay, Maria Virgínia Alves Martins, Silas Gubitoso, Leonardo Antônio Pregnolato, Laura Misailidis Lerena, Silvio Miranda Prada, José Eduardo Bevilacqua

**Affiliations:** 1 Escola de Artes Ciências e Humanidades, Universidade de São Paulo, Rua Arlindo Béttio, São Paulo, SP, Brasil; 2 Instituto de Geociências, Universidade de São Paulo, R. do Lago, São Paulo, SP, Brasil; 3 UMR 7159, IPSL/LOCEAN, Centre IRD France Nord, Bondy Cedex, France; 4 Universidade do Estado do Rio de Janeiro, Faculdade de Geologia, Departamento de Estratigrafia e Paleontologia, Av. São Francisco Xavier, Maracanã, Rio de Janeiro, RJ, Brazil; 5 Universidade de Aveiro, GeoBioTec, Departamento de Geociências, Campus de Santiago, Portugal; 6 Centro de Estudos Químicos. UNIFIEO—Centro Universitário FIEO, Av. Franz Voegeli, Bloco Branco, 4º, andar, Osasco, SP, Brazil; 7 Companhia Ambiental do Estado de São Paulo—CETESB, Av. Prof. Frederico Hermann Jr., São Paulo, SP, Brazil; Universidade de Aveiro, PORTUGAL

## Abstract

*The Dutos e Terminais do Centro Sul* (DTCS) is one of the largest petroleum terminals of the South America located in the São Sebastião Channel (SSC) on the southeastern Brazilian coast. The aims of this study were to compare the sediment quality near the DTCS with that of several sites in the SSC region including the Araçá (AR) domestic sewage outfall and to assess the efficiency of the DTCS wastewater treatment plant. To achieve these goals, textural, geochemical, and living benthic foraminifera results were analyzed for the DTCS, AR, and SSC regions. Sediments in the DTCS area were silty with high concentrations of total organic carbon (1.7–2.4%), total nitrogen (0.2–0.3%), total sulfur (0.4–0.6%), and total (0.12–0.18%) and inorganic phosphorous (0.07–0.11%). These values were higher than those in sediments collected in the SSC and Araçá regions. The sediments’ concentrations of As, Cd, Cr, Cu, Hg, Ni, Pb, and Zn in the SSC and AR regions were lower than their corresponding probable effect levels (PELs). However, sediments near the DTCS were enriched with As, Cu, and Ni, whose concentrations exceeded their corresponding threshold effect levels (TELs). Around the DTCS outfall diffusers, living foraminiferal densities and diversities were lower than those for the other areas studied. In the DTCS area, it was necessary to search 50 to 190 cm^3^ of sediment to find 100 live specimens. In the SSC and Araçá areas, a maximum of 40 cm^3^ of sediment was enough to locate 100 live specimens. The lower density and diversity of living foraminifera around the DTCS than around the other areas illustrates the impact of the environmental stress caused by the presence of pollutants. These results indicate that the wastewater treatment plant efficiency is low and its discharge of pollutants from petrochemical waste liquids affects the benthic fauna around the DTCS in a potentially harmful manner.

## Introduction

Aquatic environments, including coastal areas, have been affected by the exponential increase in human activities during the last century. Anthropogenic contaminants are introduced into the water column in different forms. For example, heavy metals, which are a major source of contamination, may be introduced in particulate, colloidal, or dissolved forms [1]. In the water column, they are adsorbed onto particles, after being deposited and accumulating in sediments. However, owing to desorption and remobilization processes, heavy metals may be released from sediments into interstitial waters and into the water column [2], [3], [4]. Sediments therefore may act either as sinks for or as sources of heavy metals. Therefore, the evaluation of metal distribution in surface sediments is important when assessing the degree of pollution in a marine environment [5].

Historically, documenting physicochemical changes in sediments has been the dominant method for determining pollution impact [6], [7]. Chemical analyses of the sediment have been conducted to assess the type and concentration of pollutants [8], [9], [10]. However, these techniques have limitations as they provide information regarding only some pollutants and exclude a wide variety of parameters, namely the responses of living organisms to increased environmental stress caused by pollution [11], [12], [13], [14].

Among the taxa used for biomonitoring methods in coastal environments, benthic foraminifera are one of the most commonly used groups owing to their abundance and diversity in marine sediments, their small size, and their preservation potential [11], [15], [16]. Since the first mention of the effect of pollution on foraminifera [17], numerous studies have shown that foraminifera are sensitive to contaminants and are effective indicators of the impact of pollution on marine and estuarine ecosystems. Although they are not included in the official protocols for environmental characterization, they increasingly are being used in environmental quality assessment, and their advantages have been widely demonstrated in the literature [11], [12], [13], [18], [19], [20], [21], [22], [23], [24], [25], [26].

Yanko et al. [27] and Nigam et al. [28] have summarized the responses of foraminifera to several types of pollution, including domestic and industrial pollution. However, deciphering the impacts of domestic and industrial pollutants is difficult because they often occur together in sheltered coastal environments (bays or estuaries). When they occur separately, it is often in environments with different natural conditions, which makes comparison problematic. The area selected for the present study, the São Sebastião Channel (Brazil), is an open area where industrial and domestic effluents are separately disposed but under similar natural conditions, offering the opportunity to compare their impact on benthic biota.

Another difficulty in using this group is the reduced knowledge of foraminifera ecology in Brazilian coastal environments, as almost all the studies have been based on the total assemblages of these organisms, that is, on the total number of living and dead organisms (e.g. [29], [30], [31]). Some exceptions are the works of Teodoro et al. [25], Duleba & Teodoro. [32], Delavy et al. [33], and Martins et al. [34], [35]. Dead or total assemblages (dead+living) do not allow rigorous inferences to be made on the behavior of species as a function of seasonal environmental variables.

### Main goals of the work

The objectives of this study were to i) evaluate the environmental impact of the largest petroleum terminal in Brazil based on living benthic foraminifera and ii) compare this industrial influence to the impact caused by neighboring domestic wastewater discharge on these organisms. The concentrations of trace elements, ejected in both effluents, will be used as an indicator of the extension and intensity of the chemical impact around both outfalls and compared with the sediment quality assessment guidelines that are being used in Brazil.

Thus, this study is one of the few carried out in Brazil that analyzes the response of living organisms (foraminifera) to effects caused by anthropogenic impacts. It can be an important contribution to a better understanding of the ecology of this group and of their behavior in a tropical environment.

## Study area

**Fig 1:**
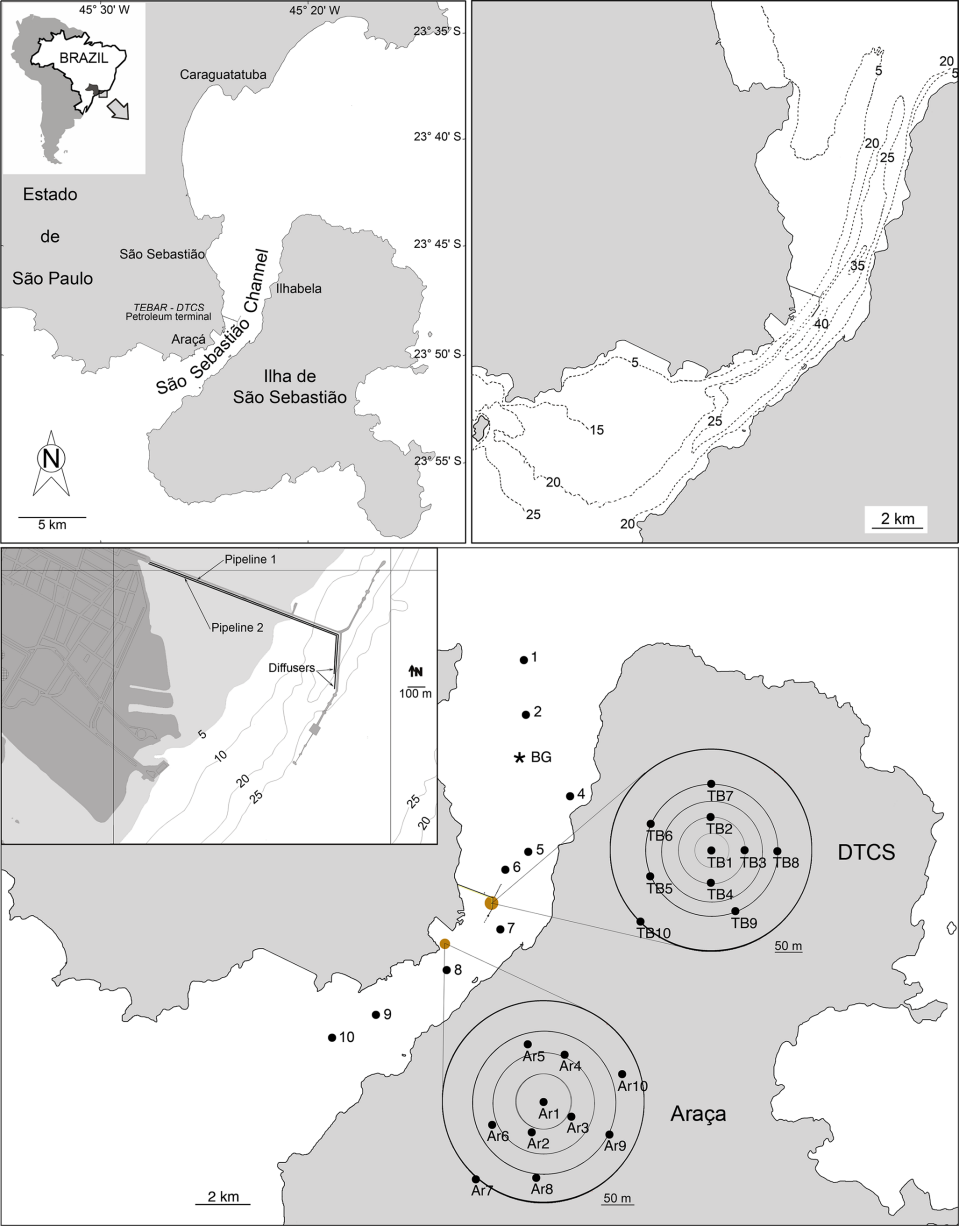
Study areas and sampling grid (SE coast of Brazil).

This study concerns the São Sebastião Channel (SSC) that lies between latitudes 23° 40′ S and 23° 53.5′ S and longitudes 45° 19′ and 45° 30′ W. It is a 25-km-long channel that separates the mainland from São Sebastião Island (Fig 1). Its width ranges from 2 km in the central portion to 7 km for both its southern and northern ends. Because of erosive processes and its bottom structure, the greatest depths (30 to 50 m) occur closer to the island (Fig 1). A wide shallow area (<5 m) exists on the mainland side of the channel. At the southern and northern ends of São Sebastião Channel, the depths are 25 and 20 m, respectively. In the SSC, emphasis was on two polluted areas: the Araçá region, a source of domestic pollution; *Dutos e Terminais Centro Sul* (DTCS, also named Oil *Terminal Almirante Barroso*–TEBAR), the largest petroleum terminal in this country and a source of industrial pollution.

Currents inside the channel move alternately northward and southward, with a periodicity of several days, owing to changes in wind patterns [36]. The shape and bathymetry of the channel produce stronger longitudinal currents on the island side, up to 1.0 ms^-1^ northward and 0.7 ms^-1^ southward [37]. The main water masses of the SSC are Coastal Water (CW; temperature >20°C, salinity <35), South Atlantic Central Water (SACW; temperature between 9°C and 18°C, salinity between 34.5 and 36), and Tropical Water (TW; temperature <20°C, salinity >36.4) [38]. The vertical density structure of the water column has a strong seasonal signal: in the winter, the waters are almost homogeneous (CW or CW/TW), whereas during the summer, a strong thermocline frequently occurs between the mid-depths and the bottom (CW/SACW).

The distribution of sediments within the SSC is related to the shape and bathymetry of the channel and to local circulation. The shape of the channel, specifically its lateral curvature, resembles a river channel with a depositional side (the mainland) and an erosional side (the island). The morphology of the bottom is extremely irregular, with great variations in depth over short distances, leading to a patchy distribution of sediments and great variations in grain size.

Some areas inside the SSC are subjected to strong anthropogenic inputs. The central region of the channel is the most affected, owing to the presence of the São Sebastião Harbor, the Araçá domestic sewage submarine outfall, and the DTCS [39] [40] [41] [42]. The DTCS comprise two piers with four berths and is operated by Petrobras Transporte SA (Transpetro). The DTCS operates with tankers carrying national and imported petroleum and derivates. TEBAR began operations in 1967. In 2006, 94% (approximately 46 million m^3^) of the oil and petroleum derivatives loaded at this terminal was transported via maritime transportation and 6% via oil pipelines, whereas 90% of all products unloaded at this terminal was transported via oil pipelines and 10% via petroleum tankers [39].

The DTCS generates two types of liquid effluents. The first type consists mostly of water, separated from oil by density during transportation from drilling platforms in the oil tankers; the second type consists of rainwater and industrial water from the DTCS, contaminated by oil. These waters are treated in the wastewater treatment plants (ETE—*Estação de Tratamento de Efluentes*), where they are first separated from residual oil by adding a solution of polyelectrolyte [40]. Then, a series of treatments, using hydrogen peroxide at several pH levels, allows the oxidization of sulfides and phenol, before an ultimate neutralization of the effluent. Approximately 15,000 m^3^ of produced waters are treated every month [41]. According to Fortis et al [40] contaminated rainwater and industrial water are treated in systems that use mostly decantation to separate oil from water (SAO = *Separação de Águas Oleosas*). In addition, if the quality of the outflowing water does not satisfy the legal regulations, it is sent for treatment in the ETE. After treatment, effluents from both the ETE and SAO are mixed before being discharged through two submarine pipelines, one each 1600 and 1400 m long, each ending in diffusers comprising three vertical, 1.5-m-long pipes, 0.15 m in diameter, located at a depth ranging from 20 to 25 m [40] (Fig 1).

The oil separation techniques, used in the wastewater treatment plants, primarily remove particulate matter and dispersed oil, while dissolved hydrocarbons remain in the discharged water [40], [43], [44]. The treated water is generally enriched with ammonia [40] and dissolved ions of sodium, potassium, magnesium, chloride, and sulfate, leading to salinity levels of up to 52.8 [40], [44]. Fortis et al [40] found in the DTCS effluent high concentrations of ammonia (125.5 mg L^-1^). The treated water also has elevated levels of some heavy metals, as well as corrosion and scale inhibitors, biocides, dispersants, emulsion breakers, and other chemicals [45], [46].

Since 1967, when operations of the DTCS commenced, numerous oil leakages have occurred [47]. Resultantly, hydrocarbons are present almost everywhere in the sediment around the SSC, although, sometimes in low concentrations [47].

## Material and methods

### Ethics statement

The study was realized with the permission of the Environmental Agency of the State of São Paulo (CETESB—Companhia Ambiental do Estado de São Paulo), Brazil, the authority that evaluate the adequacy of the waste water plants projects, the definition of environmental monitoring programs and the regulation and enforcement of the water quality compliance. The field studies did not involve endangered or protected species and areas, or particular spaces. No vertebrate sampling was conducted and therefore no approval was required by the Institutional Animal.

### Sample collection and handling

Sediment samples from DTCS (named TB) were collected near the outfall diffusers in September 2005, by CETESB. The sampling grid, consisting of 10 sampling points, was located in an area of 125.000 m^2^ surrounding the diffusers. In addition, samples from 10 stations along the São Sebastião Channel (SSC) and 10 stations around the Araçá (AR) domestic sewage outfall were used for comparison. The station locations in the TB, AR and, SSC areas are presented in S1 Appendix. Textural and biotic data from the Araçá area were previously studied by Teodoro et al. [25]. Trace elements, however, were acquired during the current study, as were all the other analyzed data from SSC and DTCS.

The geographical positions of the sampling stations were determined using the Global Positioning System (GPS), with the UTM datum SAT 69.

A Petersen open top grab sampler was used. It was modified with the addition of two metal sheets that hermetically closed the sampler for preventing unconsolidated surface sediment from being washed away during collection. Surface sediment samples were collected for the following analyses: (i) grain size analyses, (ii) geochemical analyses, and (iii) determination of living benthic foraminifera. Potential redox (Eh) and pH were measured on board, immediately after sampling, using a *Beckman Zeromatic* pH meter. The surface of the sediment was intact and the sedimentary column was undisturbed. The top 2 cm of undisturbed sediment were collected for sedimentological and foraminiferal analyses. Sediments for geochemical studies were sealed in polyethylene bags and frozen.

### Grain size and geochemical analyses

Grain size analyses were performed according to standard sieve and pipette methods. Sediments were classified according to the textural class frequencies identified by Wentworth [48] and statistical parameters identified by FoIk and Ward [49].

For geochemical analyses, sediments were dried at 60°C for 18 h. Shell fragments greater than 0.5 cm in size were removed before the sediment was homogenized in an agate mill.

Calcium carbonate concentrations were determined using weight loss after digestion with HCl [50]. Sediments were weighed (40 g), digested with 10% HCl for 48 hours, washed with distilled water, and dried at 60°C and then weighed again [50]. Weight differences corresponded to the carbonate contents in each sample.

Total organic carbon (TOC) content was measured using a LECO CNS 2000. To eliminate carbonates, 100 mg of sediment was separated and maintained for 48 h in 10% HCl [25]. The samples were then washed with distilled water and dried in an oven at 60°C. Aliquots of 0.5 g were separated and introduced into the analyzer. Total nitrogen (TN) and total sulfur (TS) were also analyzed with the LECO CNS 2000 in aliquots of 0.5 g of bulk sediment (without removing carbonates).

Organic P (OP) was estimated from the difference between the HCl-extractable P of ignited (300°C) and unignited samples [51]. The fractionation of inorganic P (IP) was determined using the method presented by Legg and Black [51].

Elemental TOC/TN (C/N ratio) and molar TOC/OP (C/OP) ratios were calculated to determine the source and nature of organic matter in the surface sediments. The elemental TOC/TS ratio (C/S ratio) was also considered a qualitative indication of the redox status of the sediments and overlying water column [52], [53].

Trace elements were investigated in aliquots of the entire homogenized sample that had been reduced to a fine powder using an agate mill. The analyses were performed by ACMELABS, Canada. A modified *aqua regia* solution of equal parts concentrated ACS-grade HCl and HNO_3_ and demineralized H_2_O was added to each sample (6 mL g^-1^) for leaching in a hot water bath (~95°C) for one hour. After cooling, the solution was brought up to its final volume (20 mL g^-1^) with 5% HCl. The concentrations of arsenic, barium, cadmium, cobalt, chromium, copper, lead, mercury, nickel, scandium, and zinc were determined using ICP-MS (Perkin Elmer Elan 9000).

#### Sediment quality and evaluation methods of anthropogenic impact

Sediment quality criteria are among the major management tools used in the maintenance of acceptable conditions for living resources. Therefore, numerical sediment quality assessment guidelines have been established to help in the assessment of sediment contamination. The Florida Department of Environmental Protection (FDEP) [54] published the “Development and Evaluation of Sediment Quality Assessment Guidelines,” in which two levels of contamination were defined: the threshold effect level (TEL), which is the concentration at which effects rarely occur, and the probable effect level (PEL), which is the concentration at which effects are likely to occur. These levels of contamination were established for arsenic, cadmium, chromium, copper, lead, mercury, nickel, silver, and zinc.

Long et al. [55] established two guideline values for these trace metals. The two values, i.e., effects range-low (ERL) and effects range-median (ERM), define concentration ranges that are rarely (< ERL), occasionally (between ERL and ERM), or frequently (> ERM) associated with adverse effects. The values of ERL and ERM are notably higher than TEL and PEL, respectively. For example, for As: ERL = 8.2 mg kg^-1^ and TEL = 7.24 mg kg^-1^; ERM = 70 mg kg^-1^ and PEL = 41.6 mg kg^-1^.

Later, the Canadian Council of Ministers of the Environment (CCME) [56] [57] published the Canadian sediment quality guidelines for the protection of aquatic life, defining the interim marine sediment quality guidelines (ISQG) and the probable effect level (PEL). The values of the ISQG are the same as those of TEL and PEL of the FDEP [54]. In Brazil, the *Conselho Nacional do Meio Ambiente* (National Council for the Environment [58]), by its resolution CONAMA 344/2004, assumes the values established by Long et al. [55] for defining the two levels of contamination. In this study, the concentration of trace metals in sediment was compared to the two levels of contamination established by CONAMA [58], FDEP [54] and CCME [56], [57].

To assess sediment quality and degree of anthropogenic input, the metal enrichment factor (EF) [59] [60] [61] and the index of geoaccumulation (Igeo) [62] were used.

The EF compares the ratio of the concentration of one element from the studied sample to the concentration of a reference element in the same sample, used for normalization, with the same ratio calculated at a reference station (SSC3), which is considered uncontaminated, using the following equation: EF = [(Cx/Cref)]_Sample_/[(Cx/Cref)]Background—Eq 1, where Cx is the concentration of the element of interest (mg kg^-1^), and Cref is the concentration of the reference element (mg kg^-1^) for normalization. The significance of the EF values is summarized in Table 1 from Sutherland et al. [59].

**Table 1:** Enrichment factor values and their significance.

EF	Significance
**<2**	Deficiency to minimal enrichment
**2–5**	Moderate enrichment
**5–20**	Significant enrichment
**20–40**	Very high enrichment
**>40**	Extremely high enrichment

In the current study, the element used for normalization was scandium, which is not used in industrial activities and is mostly of crustal origin [60]. Among the stations of the São Sebastião Channel, SSC3, located far from the diffuser (at about 4 km) and with low concentrations of contaminants, was selected as the reference station (RS) (Fig 1).

The Igeo assesses the degree of metal pollution in terms of seven enrichment classes based on the increasing numerical values of the index [62] (Table 2). This index is calculated using the following equation: Igeo = log_2_ (Cn/1.5*Bn)—(Eq 2), where Cn is the concentration of the element in the enriched samples, and Bn is the background or pristine value of the element, measured at the reference station (SSC3). According to Stoffers et al. [63] and Abrahim and Park [61], the factor 1.5 is introduced to minimize the effect of possible variations in the background values, which may be attributed to lithological variations in the sediments.

**Table 2:** Müller’s geoaccumulation index defining seven classes of sediment quality [62].

Igeo value	Igeo class	Designation of sediment quality
**>5**	6	Extremely contaminated
**5–4**	5	Strongly to extremely contaminated
**4–3**	4	Strongly contaminated
**3–2**	3	Moderately to strongly contaminated
**2–1**	2	Moderately contaminated
**1–0**	1	Uncontaminated to moderately contaminated
**<0**	0	Uncontaminated

### Foraminiferal analysis

Immediately after sampling, the samples used for the identification of foraminifera were fixed with 70% alcohol stained with 1 g Rose Bengal, to distinguish stained (living) from unstained (dead) benthic foraminifera [64]. Aliquots of 10 cm^3^ of sediment were washed through two sieves: 0.5 and 0.063 mm [65]. The obtained fractions were dried, and the foraminifera were separated from the sediment by flotation using trichloroethylene. In samples with a low number of foraminifera, aliquots of 10 cm^3^ were successively analyzed for a count of at least 95 stained individuals, the lowest foraminifera quantity considered feasible for statistical analysis [66], [67]. Therefore, about 95 or more stained foraminifera were handpicked for identification and counting at each station. Foraminiferal density 1 (Density 1) is expressed as the number of foraminifera per volume of sediment and Density 2 is number of foraminifera per 10 cm^3^ [68]. The richness is defined as the total number of species found at each station. Richness values were calculated from Density 1.

### Statistical analysis of abiotic and biotic data

In terms of foraminiferal assemblages, specific diversity was determined using the Shannon index (H') [69]. The equitability (J) was calculated according to the Pielou index [70].

Canonical correspondence analysis (CCA) was used to investigate the relationship between foraminifera and sedimentological variables of the three areas (TEBAR, Araçá, and the São Sebastião Channel). The analysis was done using CANOCO 4.5 software [71]. Only species with more than 40 specimens in the entire dataset were included in the biological matrix [72], [73]. Rare species were eliminated because they can produce noise in CCA [74]. Abiotic and biotic data were transformed using ln(x + 1) to increase the importance of smaller values, leading to a more normalized distribution [75].

The pattern emerging from this matrix (16 species x 29 samples) was related via CCA to 23 variables contained in the hydrographic/sedimentological matrix. The environmental variables were: depth, bottom salinity, bottom temperature, bottom dissolved oxygen,% sand,% mud, C/N and C/S ratios, TOC, TP, TN, TS, As, Ba, Cd, Co, Cr, Cu, Hg, Ni, Pb, Sc, Sr, and Zn.

The Monte Carlo permutation test (999 permutations) was used to assess the statistical significance of the correlations (at *p* < 0.05 and *p* < 0.01). On the biplot produced from this analysis, eigenvectors showed the axes and direction of increasing values for each environmental factor. The length of each eigenvector reflected the correlation between the factor and the meiofaunal pattern. The ranking of a species or sample relative to each environmental variable was determined by projecting its plotted position perpendicular to the appropriate eigenvector.

## Results

Sedimentological, hydrographic and biotic data are summarized in Appendices 1–3 and are presented in the following items.

### Physicochemical properties of water

#### São Sebastião Channel

Samples were collected at depths ranging from 2.5 to 13.9 m. The temperature and salinity values in the water column oscillated from 20.1 to 24.1°C and from 36.0 to 37.2, respectively (S1 Appendix). Weak thermohaline stratification was observed at all stations excluding the reference station (SSC3; Fig 1), where the water column was homogeneous. The pH ranged from 7.95 to 8.08, and dissolved oxygen ranged from 4.81 to 7.72 mg L^-1^. Turbidity varied from 0.55 to 3.10 NTU.

At the reference station (SSC3), temperature and salinity values of 24.0°C and 36.7°C, respectively, were observed. The pH and dissolved oxygen values varied from 8.06 to 8.07 and from 6.84 to 6.87 mg L^-1^, respectively. Turbidity varied from 2.3 (at the bottom) to 3.1 NTU (at the surface).

#### Dutos e Terminais Centro Sul (DTCS)

Samples were collected at depths ranging from 10.5 to 31.5 m. The water column throughout the DTCS area was typically weakly stratified. The temperature and salinity values in the water column ranged from 21.3 to 24.3°C and from 34.8 to 35.7, respectively. The pH and dissolved oxygen values varied from 8.15 to 8.18 and from 6.34 to 7.35 mg L^-1^, respectively. Turbidity oscillated between 1.3 and 5.3 NTU.

#### Araçá outfall

Samples were collected at depths between 3.5 and 17.5 m. The water column at the Araçá outfall did not present significant stratification. Temperature and salinity values in the water column ranged from 22.1 to 23.3°C and from 34.6 to 35.1, respectively. The pH and dissolved oxygen values varied from 8.17 to 8.24 and from 6.33 to 7.60 mg L^-1^ respectively. Turbidity values oscillated from 0.71 (at the bottom) to 4.30 NTU (at the surface).

### Surface Sediment Texture in São Sebastião Channel (SSC)

**Fig 2:**
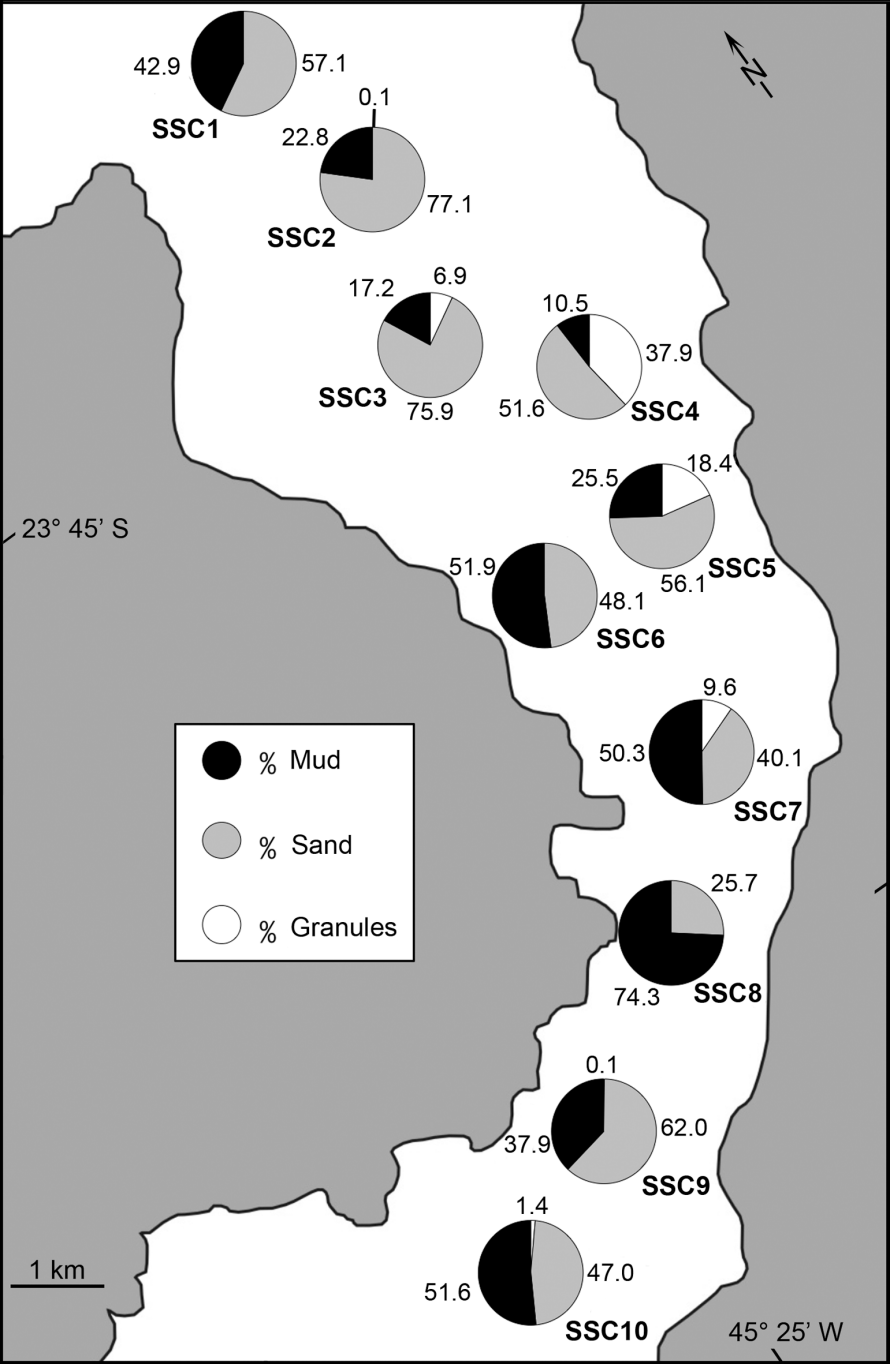
Sand, mud and granule percentage in samples collected along the São Sebastião Channel.

The mean sediment grain size ranged from 0.6 to 5.8 Ф (S1 Appendix). The grain sorting varied between poorly and extremely poorly sorted (δ = 1.6–4.8). Sandy sediments were predominant at stations SSC1–SSC5 (in the northern area), with the sand fraction comprising 51.6% to 77.1% of the sediment (Fig 2). The mud fraction was present at higher concentrations in the central (SSC6–SSC8) and southern (SSC10) areas of the channel, reaching 74.3% at station SSC8. Large fragments of gravel-sized mollusk bioclasts were observed in significant percentages at stations SSC4 (37.9%) and SSC5 (18.4%) (S2 Appendix). Sediments of the reference station (SSC3) were sandy (51.6%) with poor grain sorting.

#### Dutos e Terminais Centro Sul (DTCS)

**Fig 3:**
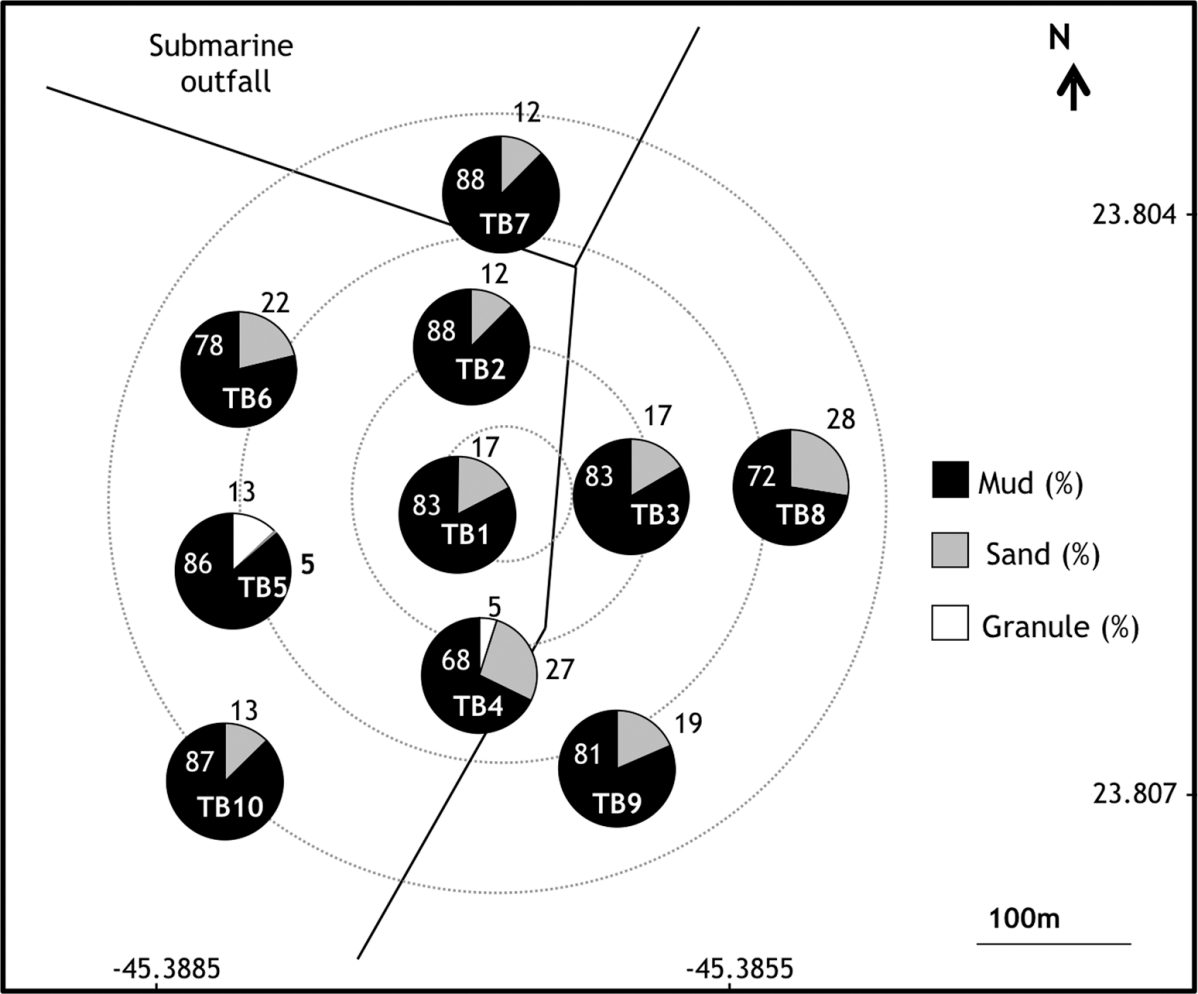
Sand, mud and granule percentage in samples of the DTCS (*Dutos e Terminais Centro Sul* or Oil *Terminal Almirante Barroso–*TEBAR; TB), diffusers.

Sediments near the DTCS submarine outfall diffusers were composed primarily of the mud fraction (67.8–87.6%) (Fig 3). The coarse fraction, which reached a significant percentage at station TB5 (13.1%), was made up of gravel and/or mollusk bioclasts. Sediment sorting ranged between poorly and very poorly sorted (δ = 1.5–3.9).

#### Araçá outfall

The sediment mean grain size and grain sorting ranged from 1.8 to 4.1 Ф and from 1.6 to 3.2 δ (poorly to very poorly sorted), respectively [25]. The sandy fraction was predominant (46.4 to 82.7%). Significant mud content was observed at station AR10 (53.2%; S2 Appendix). Gravel (fragments of mollusks) occurred at almost all of the stations, with percentages ranging from 0.4 to 12.6%.

### Surface Sediment Geochemical Composition of São Sebastião Channel (SSC)

**Fig 4:**
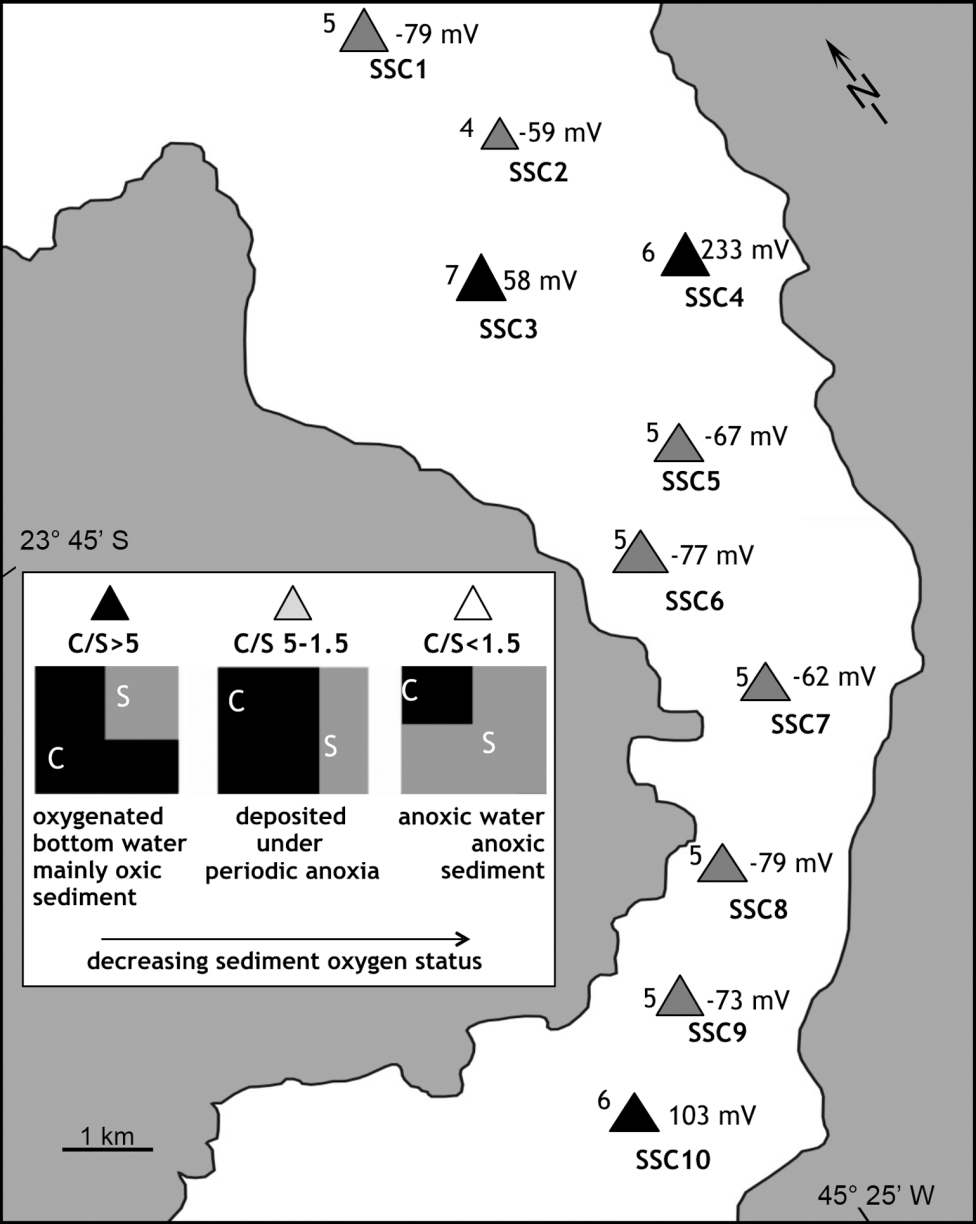
Values of Eh and C/S (TOC/TS) ratio of samples collected along the São Sebastião Channel (SSC). The meaning of C/S ratio values (modified from http://www.ozcoasts.gov.au) is also presented. Symbols are proportional to C/S ratio values.

The pH and redox potential (Eh) ranged from 6.9 to 7.7 and -79 to 233 mV, respectively. Positive redox potential was observed only at stations SSC3 (RS), SSC4 and SSC10 (Fig 4).

All sediments were essentially lithoclastic, with CaCO_3_ <30%. The TOC content varied from 0.39% to 2.67% (S2 Appendix). The highest values of TOC were observed at stations SSC6–SSC8. The TN concentrations presented an almost homogeneous distribution, ranging from 0.20% to 0.29%, except at stations SSC1 (0.13%), SSC2 (0.17%), and the reference station (SSC3; 0.09%). The concentrations for TP ranged from 0.017 to 0.055%, for OP from 0.002 to 0.023%, for IP from 0.015 to 0.038% (S2 Appendix). The TS concentrations ranged from 0.07 to 0.49%, with the highest values observed at stations SSC6-SSC8.

**Fig 5:**
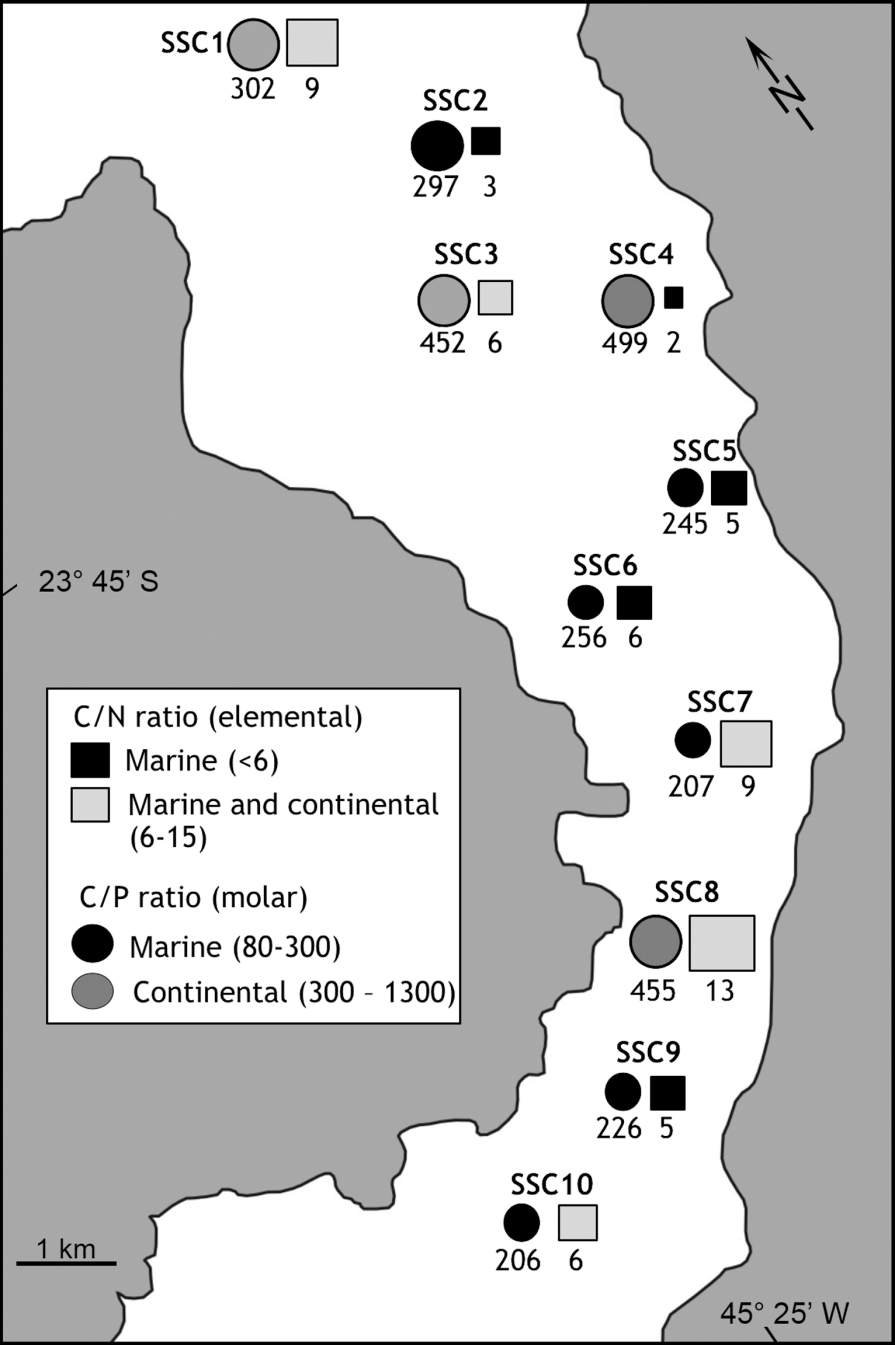
C/N ratio and C/OP ratio of the São Sebastião Channel. Symbols are proportional to the values.

The sediments collected from the reference station presented low TOC, TS, and nutrient concentrations and positive Eh values. The values of the C/N (elemental) and TOC/OP (C/OP; molar) ratios ranged from 2 to 13 and from 206 to 499, respectively (Fig 5). TOC/TS (elemental) ratios ranged from 4 to 6, with the highest values observed at stations SSC3 (7), SSC4 (6) and SSC10 (6) (Fig 4).

The highest trace element concentrations were found in the central part of the channel, particularly at station SSC8, which is located a short distance from the Araçá outfall, and at station SSC10, where Ba reached its highest value. The lowest trace element values were found at stations SSC4 and SSC3 (RF). Trace element concentrations were higher in the southern part of the channel than in the northern part.

#### Dutos e Terminais Centro Sul (DTCS)

The pH varied between 6.5 and 7.4, with the minimum value observed at station TB3 and the maximum value observed at station TB9. The Eh of the surface sediments ranged from -171 to -81 mV.

**Fig 6:**
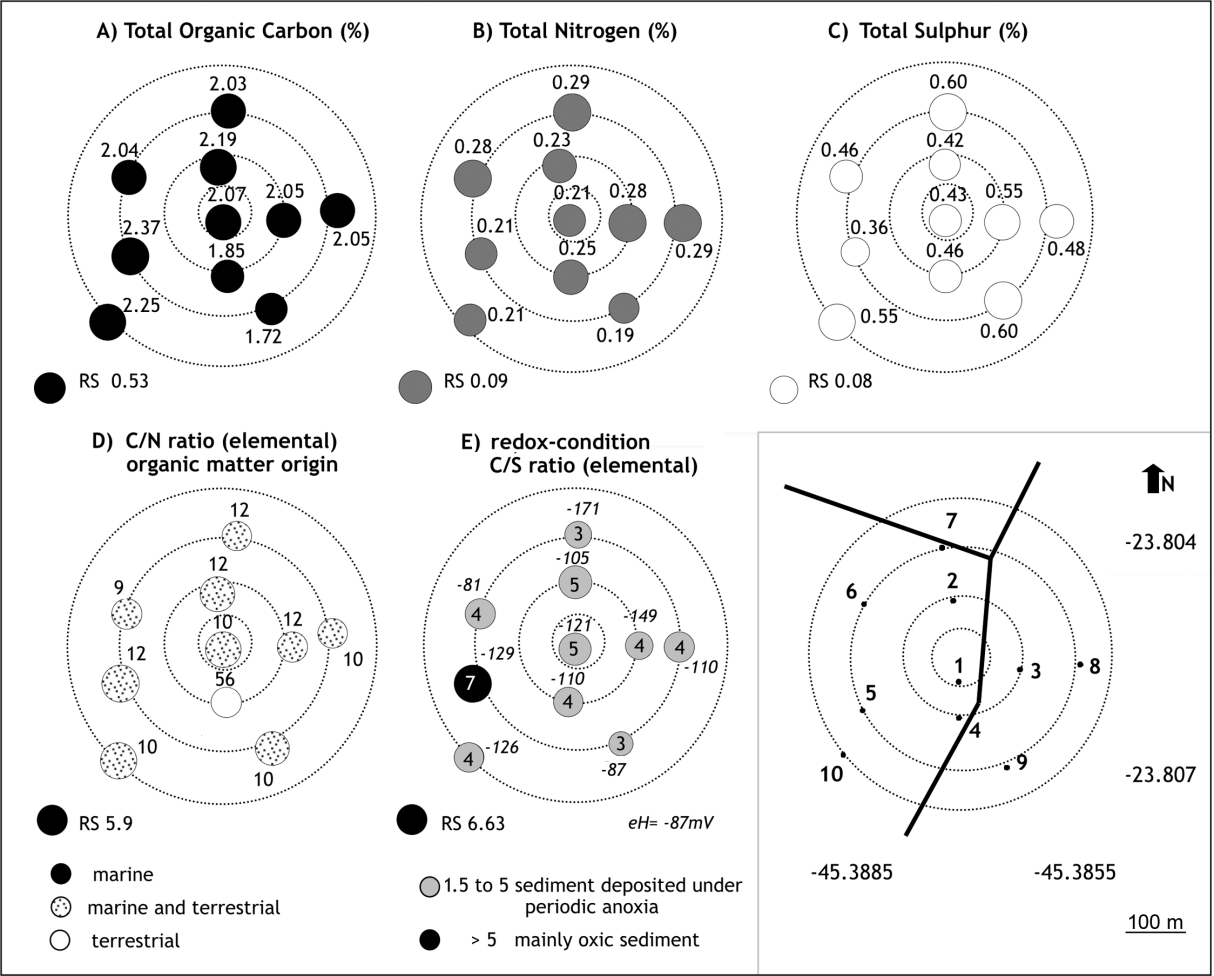
Concentrations of total organic carbon (%), total nitrogen (%), total sulfur (%), C/N and C/S ratios of DTCS—TEBAR (S2 Appendix). Different infilling and colors of the symbols represent different ranges of values. RF: reference station.

For the DTCS stations, the sediments were also lithoclastic (CaCO_3_ <30%). The concentration ranges for different parameters were as follows: TOC (Fig 6A) from 1.72 to 2.37% (in TB9 and TB5, respectively); TN from 0.19 (TB9) to 0.29% (TB7) (Fig 6B); TS from 0.36 (TB5) to 0.60% (TB7, TB9) (Fig 6C).

**Fig 7:**
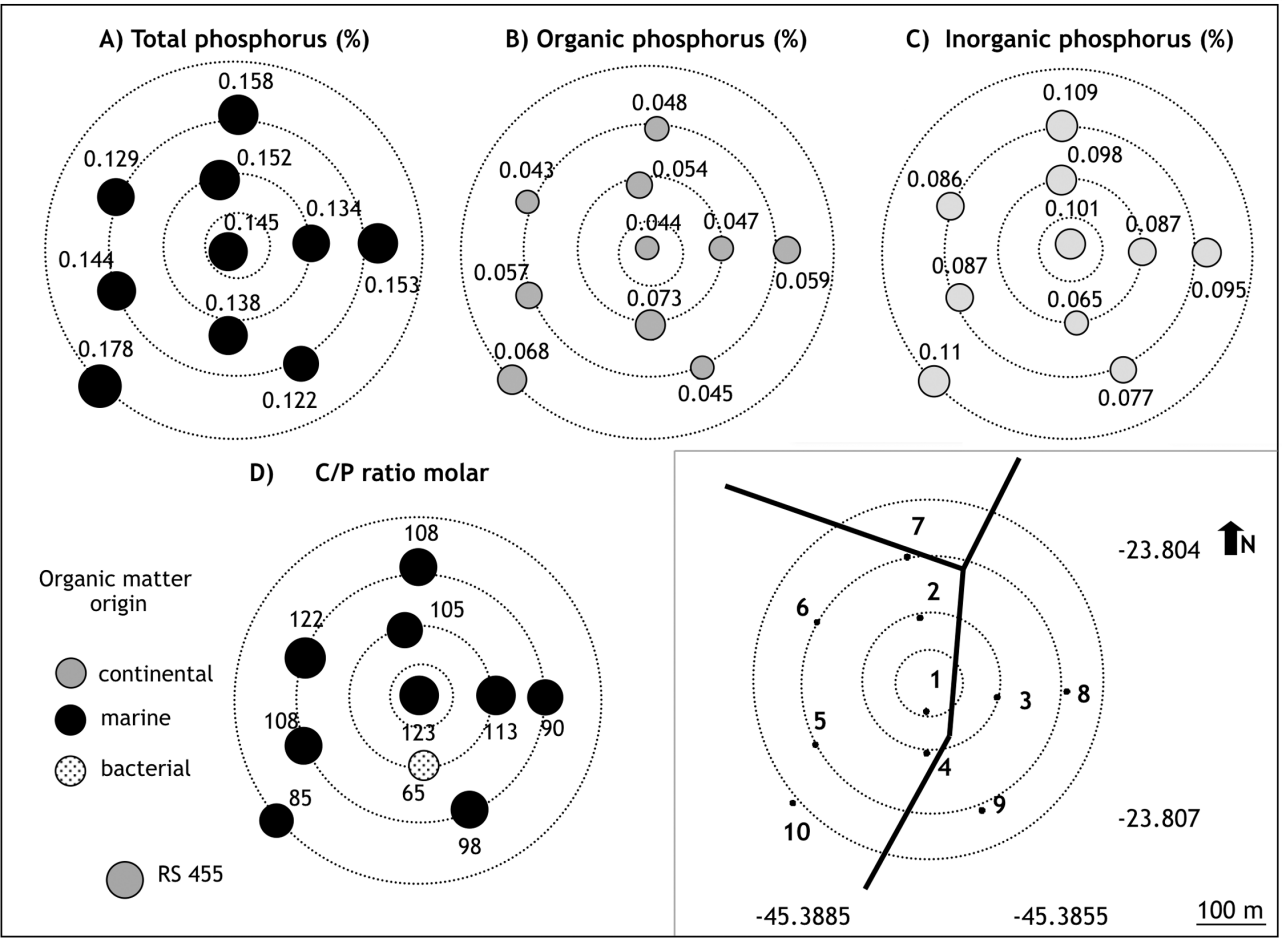
Concentrations of total phosphorus (%), total organic phosphorus %), total inorganic phosphorus (%), C/OP ratio (molar) of TEBAR (S1 Appendix). Different infilling and colors of the symbols represent different ranges of values. RF: reference station.

The concentrations for TP ranged from 0.12 (TB9) to 0.178% (TB10) (Fig 7A), for OP from 0.043 (TB6) to 0.07% (TB4), with the highest concentrations at stations TB4 and TB10 (Fig 7B); and IP from 0.077 (TB9) to 0.11% (TB10), accounting for 46.61 to 69.87% of TP (Fig 7C).The TP concentrations were approximately threefold higher in the DTCS diffusers than in the SSC and were similar to the values found at Araçá [25].

The ratio of C/N varied between 9 and 56 (Fig 6D), of T/S between 3 and 7 (Fig 6E), and of C/OP between 65 and 121 (Fig 7D).

Higher concentrations of trace elements, such as As, Ba, Cd, Cr, Cu, Hg, Ni, Pb, Sr, and Zn, were found in the DTCS area than at the reference station. The average concentrations of the analyzed chemical elements were highest in the DTCS samples, except for Sr, which reached its highest concentrations at the Araçá outfall.

#### Araçá outfall

In the Araçá outfall area, TOC contents ranged from 0.10 (AR7) to 3.37% (AR9). Relatively high TOC content was also found in AR8 (3.31%). The concentration ranges of the following parameters were as follows: TN from 0.01 to 0.10%, TS from 0.09 to 0.46%, TP from 0.083 to 0.192%, IP from 0.058 to 0.159%, OP from 0.002 to 0.053%, C/N from 2 to 337, C/S from 1 to 19, and C/OP from 11 to 265. The highest value of TS was recorded in station AR10 [25].

A comparison of trace element contents with background concentrations revealed that the Araçá stations had higher concentrations of Sr, Cu, and Cd, and to a lesser extent, of Ba and As. Station AR10 had the highest values for all elements excluding Sr.

### Living benthic foraminifera

#### São Sebastião Channel

A total of 88 living species were identified in the São Sebastião Channel (S3 Appendix), belonging to the suborders of Rotaliina (63 species), Textulariina (20 species), and Miliolina (only one species). The volume of analyzed sediment needed to obtain 95 stained foraminifers ranged from 10 to 60 cm^3^. Density 1 values ranged from 95 specimens per 60 cm^3^ of sediment to 296 specimens per 10 cm^3^ of sediment. Density 2 values ranged from 16 to 296 specimens per 10 cm^3^ of sediment. The highest densities were identified at stations SSC3 (266 specimens) and SSC6 (296 specimens). Richness values varied from 12 to 33 species. The H’ and J values varied between 1.59 and 3.25 and between 0.64 and 0.93, respectively.

*Ammonia tepida* was the most abundant species in almost all samples (5–56.1%). The following species presented significant relative abundance: *Ammonia parkinsoniana* (2–19.6%), *Bolivina striatula* (0.8–11.6%), *Globocassidulina crassa* (<18.8%), *Globocassidulina subglobosa* (<10.2%), *Nonionella opima* (<9.1%), *Buliminella elegantissima* (2–8.5%), *Bolivina fragilis* (<8.5%), *Bulimina marginata* (<5.9%), *Pseudononion japonicum* (<5.8%), *Hopkinsina pacifica* (<5.3%), *Rosalina floridensis* (<6.1%), *Gavelinolepsis praegeri* (<5.5%) and *Hanzawaia boueana* (<5.1%).

At the reference station (SSC3), *Ammonia tepida* had the highest relative abundance (42.5%), followed by *Ammonia parkinsoniana* (16.2%), *Bolivina striatula* (7.9%), and *Buliminella elegantissima* (7.5%). At this station, 32 species were recognized.

#### Dutos e Terminais Centro Sul (DTCS)

Throughout this area, 45 species were identified as belonging to Rotaliina (37 species), Textulariina (6 species), and Miliolina (2 species). Foraminiferal densities ranged from 0.5 (TB9) to 25 (TB7) specimens per 10 cm^3^ of sediment. Owing to this low density, the volume of analyzed sediment needed to obtain 95 stained individuals varied from 40 to 190 cm^3^. Species richness varied from 12 to 23 per 95 foraminifera. The H' and J values ranged from 1.5 to 2.4 and from 0.56 to 0.71, respectively. Both indices presented low values, indicating low species diversity, due to the dominance of a few species.

*Ammonia tepida* was the most abundant species in all the samples (38.5–66%). The following species also had significant relative abundance: *Pararotalia cananeiaensis* (<20%), *Buliminella elegantissima* (0.9 to 11.8%), *Ammonia parkinsoniana* (<7.3%), *Cibicidoides lobatulus* (<6.6%), *Bolivina striatula* (<6.4%), *Bulimina marginata* (1–6.3%), *Bolivina ordinaria* (0.9–6%), *Bolivina compacta* (<5.5%), and *Rosalina floridensis* (<5%).

#### Araçá outfall

In this area, 51 species (S3 Appendix) were identified as belonging to the suborders Rotaliina (33 species), Textulariina (11 species), and Miliolina (7 species). Foraminiferal densities ranged from 28 to 98 specimens per 10 cm^3^ of sediment. A volume ranging from 10 to 40 cm^3^ was analyzed to obtain 95 stained individuals. Species richness values varied from 13 to 28 species. The H' and J values varied from 0.70 to 2.64 and from 0.69 to 0.85, respectively.

*Ammonia tepida* was the most abundant species in all samples of the Araçá region, with a relative abundance ranging between 24.7% and 47.3%. The following species also had significant relative abundance: *Pararotalia cananeiaensis* (1.8–17%), *Cibicidoides lobatulus* (<11.9%), *Bolivina ordinaria* (1.1–11.3%), *Rosalina floridensis* (<8.6%), *Buliminella elegantissima* (<7.8%), *Bolivina striatula* (3.2–7.7%), *Pseudononion japonicum* (<6.1%), *Globocassidulina crassa* (<6.1%), and *Ammonia parkinsoniana* (<5.4%).

### Results of statistical analyses

Among the 20 parameters used for the canonical correspondence analysis (CCA), only 6 (sand, TP, C/S, Cr, Sr, and Cd) were significantly correlated to species density, according to the Monte Carlo test.

**Fig 8:**
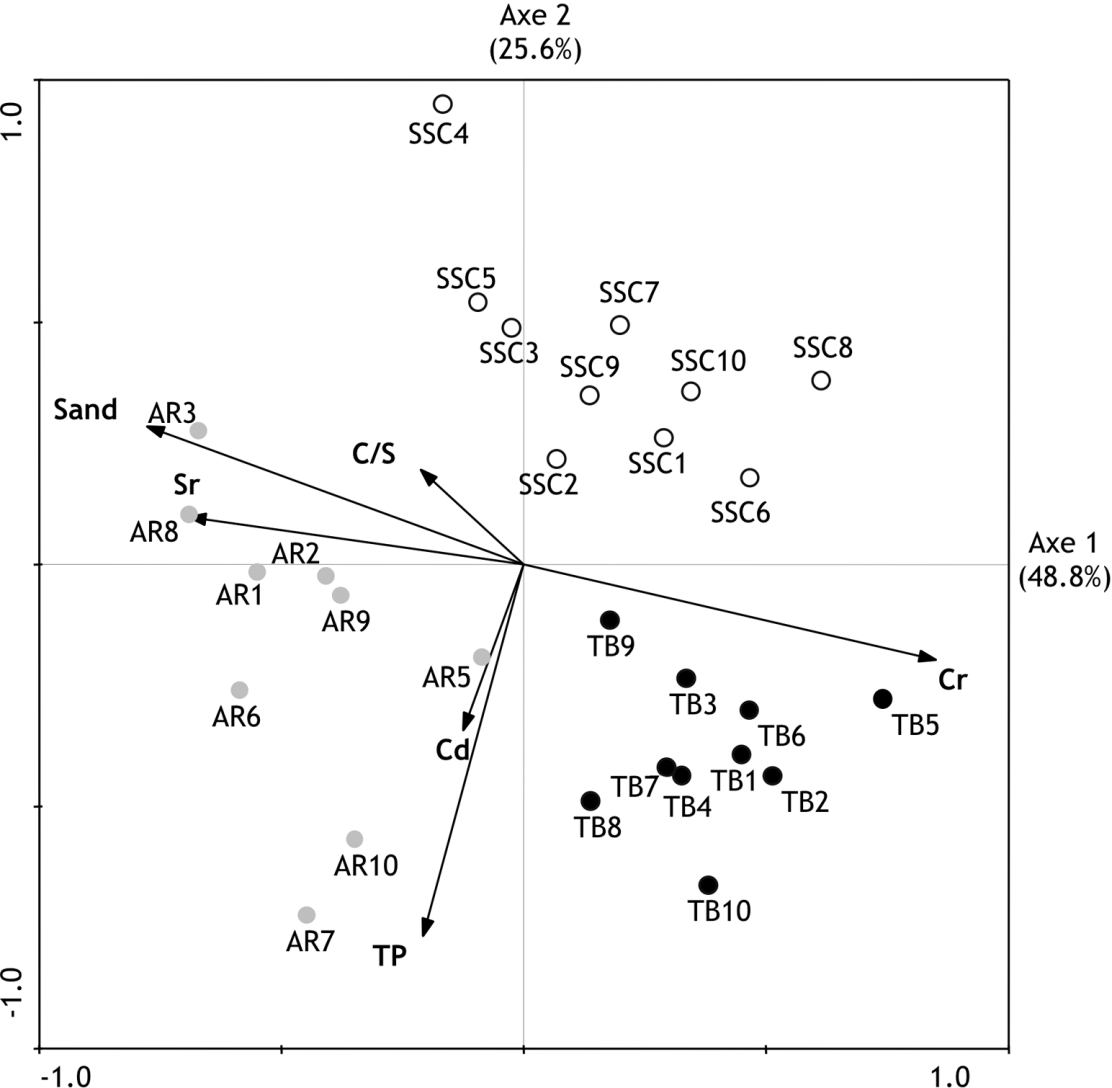
Biplot of canonical correspondence analysis based on foraminifera (species density, considering only species with more than 40 specimens in the entire dataset) and some selected sedimentological variables (sand, TP, C/S, Cr, Sr, and Cd) of the stations located in the three analyzed areas (TEBAR, Araçá, and the São Sebastião Channel).

**Fig 9:**
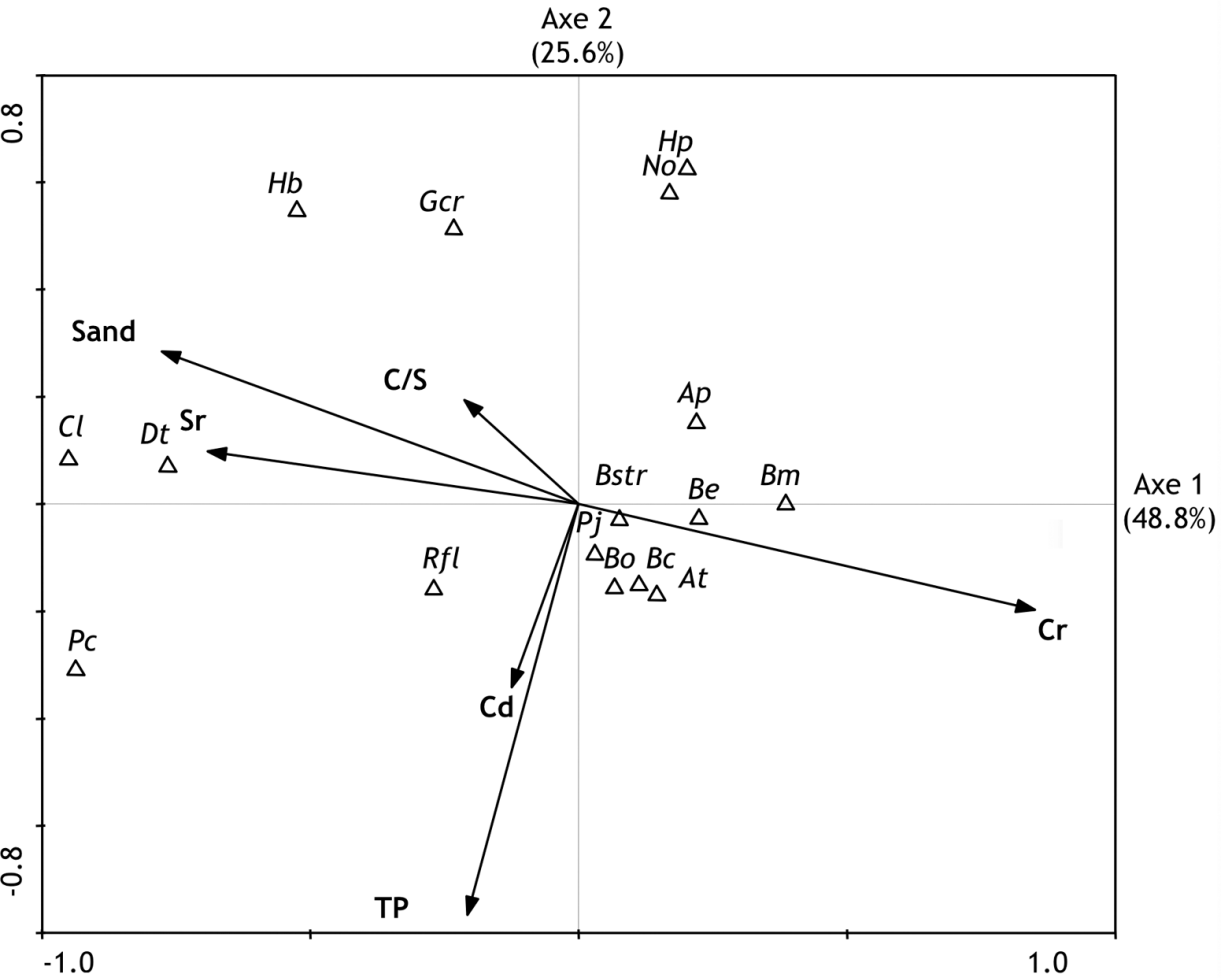
Biplot of canonical correspondence analysis of foraminiferal species (species density, considering only species with more than 40 specimens in the entire dataset) and some selected variables (sand, TP, C/S, Cr, Sr and Cd). Ap (*Ammonia parkinsoniana*), At (*Ammonia tepida*), Bc (*Bolivina compacta*), Bo (*Bolivina ordinaria*), Be (*Buliminella elegantissima*), Bstr (*Bolivina striatula*), Bm (*Bulimina marginata*), Gcr (*Globocassidulina crassa*), Hb (*Hanzawaia boueana*), Hp (*Hopkinsina pacifica*), Cl (*Cibicidoides lobatulus*), Dt (*Discorbis terquemi*), Pj (*Pseudononion japonicum*), Pc (*Pararotalia cananeiaensis*), No (*Nonionella opima*), Rfl (*Rosalina floridensis*).

The results of the CCA for Axes 1 (eigenvalue = 0.130) and 2 (eigenvalue = 0.068) explain 74.4% of the total variance of the data (Fig 8). Species–environment correlations indicated by the CCA are high, with values of 0.928 for axis 1 and 0.799 for axis 2 (Fig 9).

In the CCA of Fig 8, the parameters most significantly correlated with axis 1 in both analyses were, in decreasing order: Cr (positively correlated); sand, Sr, and C/S (all negatively correlated). The most significantly correlated parameters with axis 2 were, in decreasing order: TP and Cd, both negatively correlated. The main gradient, reflected by axis 1, is the transition from sandy, well-oxygenated sediments (i.e., highest values of C/S) with high concentrations of Sr to muddy sediments with high concentrations of Cr. The gradient reflected by axis 2 is a transition from sediments with high concentrations of TP and Cd to sediments with low concentrations of these elements.

The CCA of Fig 8 shows that most of the samples of SSC are positively related to both axes, while most of the samples of the Araçá region are negatively related to both axes, and samples from DTCS are positively related to the first axis and negatively to the second axis.

The CCA of Fig 9 illustrates that species negatively associated with axis 1 are *Cibicidoides lobatulus*, *Discorbis terquemi*, *Rosalina floridensis*, and *Pararotalia cananeiaensis*. These species are linked with sandy sediments, mostly in the Araçá area. Positive values of axis 1 are associated with *Bulimina marginata*, *Buliminella elegantissima*, and *Ammonia parkinsoniana*, which occur in muddy sediments.

The CCA of Fig 9 indicates that the species associated with positive values of axis 2 are *Hopkinsina pacifica*, *Nonionella opima*, *Hanzawaia boueana*, and *Globocassidulina crassa*, which are negatively correlated to TP and Cd accumulations. *Ammonia tepida* is plotted close to the center of the plane, towards the positive values of axis 1 and the negative ones of axis 2, and is associated with the DTCS samples. The other species are located near the center of the plane, which indicates lower impact of the analyzed parameters on these species.

## Discussion

### Physicochemical properties of the water column

Temperatures higher than 20°C (20.1–24.3°C) and salinities higher than 36.4 (36.2–37.2) in the water column of the SSC indicate, according to Castro-Filho [38], a predominance of CW mixed with TW in this area during the sampling period. Temperature and salinity values in the water column at DTCS, ranging from 21.3 to 24.3°C and from 34.8 to 35.7, respectively, suggest a predominance of CW at the sampling time. During the sampling event in the Araçá area, the salinity and temperature, ranging from 22.1 to 23.3 and from 34.6 to 35.1°C, respectively, also indicate the presence of CW.

Among the analyzed physicochemical parameters, only the dissolved oxygen from the intermediate and bottom waters at stations SSC1 and SSC6–SSC10 had values outside of the limits established by the resolution CONAMA 357/2005 [76] (< 6 mg L-1). Nevertheless, these values, even for bottom waters, were well above the conventional threshold for hypoxia (2 mg L-1), the value of 3.5 mg L-1 recommended by Steckbauer et al. [77] as the operational oxygen threshold to designate hypoxic waters, and even above the threshold of 4.6 mg L-1 considered as a «precautionary limit to avoid catastrophic mortality events» [78]. As a result, it can be inferred that the chemical characteristics measured in the water column are not limiting factors for benthic foraminiferal populations.

### Textural composition and geochemical data of the surface sediment

#### Textural composition

The SSC has a complex pattern of textural composition, with a mosaic distribution of sediment grain size that reflects the bottom topography and hydrodynamics of the channel [37]. Owing to the presence of strong currents, coarser sediments were observed on the island side with significant presence of granules at SSC4 and SSC5. Conversely, the submarine outfalls of Araçá and DTCS are positioned in areas of weaker currents, leading to the deposition of finer sediment, mostly sands around Araçá outfall, and predominantly muddy sediment in the DTCS area. The sediments found over the whole study area are in general poorly sorted, reflecting the constant change in direction and speed of bottom currents [37], which may influence the dispersion of effluents around the outfalls.

#### Potential redox and pH

The redox potential of surface sediments in São Sebastião Channel and near the DTCS outfall are mostly negative, and lower near the outfall, indicative of hypoxic conditions. Only three stations in São Sebastião Channel (SSC3, SSC4 and SSC10) have positive redox potential. According to Mitsch and Gosselink [79] and Vorenhout et al. [80], redox processes generally occur within certain boundaries of potential differences: iron and nitrate reduction can occur between +100 mV and -100 mV; sulfate reduction occurs between -100 mV and -200 mV; and, at less than -200 mV, methanogenesis processes occur. In this sense, conditions favorable for iron and nitrate reduction were observed at stations SSC1, SSC2, SSC5–SSC9, TB6, TB7, and TB9 and those for sulfate reduction at the other stations of DTCS.

The pH values for most of stations of DTCS (TB1, TB3, TB5-TB7, TB10) and stations SSC2, SSC3 (reference station), and SSC6 were below 7.0, indicating acidification in the sediment–water interface, which may be related to anaerobic OM degradation. These conditions may have affected the calcification of benthic foraminifera, as suggested by the presence of low density and fragile tests of calcareous specimens.

#### Geochemical data

Greater organic matter enrichment (High TOC content), was observed around the DTCS, at the two seaward stations around Araçá outfall (AR8 and AR9), and in the central region of São Sebastião Channel (SSC6–SSC8), located near DTCS and Araçá. It suggest that, despite a number of anthropogenic activities, such as the movement of vessels, the presence of the São Sebastião Harbor, DTCS (petrochemical wastewater) and, to a lesser extent, Araçá (domestic effluents) are the main sources of organic carbon. The TOC values measured in these stations were considerably higher than the values reported by Teodoro et al. [25] in Araçá and by Furtado et al. [37] along the insular side of the SSC (average value = 0.5%). However, they were weak compared to other areas subjected to organic pollution, such as Marmara sea or in the Firth of Clyde (Scotland), where TOC may range between 2% and 15% [81], [53]. The strong currents occurring in São Sebastian channel probably prevent organic accumulation.

In the sediments of DTCS, TN content was threefold higher than in the reference station. TS values were similar to values from marine areas under the influence of significant anthropogenic activities, such as around Santos (SE Brazil) submarine outflow (0.14 to 0.69; average 0.30 ± 0.19) [82]. TS percentages observed for TB7, TB9 and TB10 were 4 to 6 times higher than those observed in Araçá [25], 10 times higher than those of the insular side of the SSC [37], and 9 times higher than those of the reference station. These results suggest a local pollution source from the DTCS effluent discharges because sulfur is an important compound of petroleum and thus an important potential contaminant [83]. The input of sulfide results from the insufficiency of the treatment system in the DTCS that may release untreated effluents containing up to 191 mg L-1 of sulfide, according to a report of the *Companhia de Tecnologia de Saneamento Ambiental* [84]. The effluents result from the washing of tanks and the release of ship ballast waters containing a high quantity of petroleum residuals, as well as detergents used for washing (e.g., linear alkylbenzene sulfonate or dodecylbenzenesulfonate) that subsequently are discharged through the outfall [25] [85].

The TP values were higher than 1200 μg g-1 in the stations around DTCS diffusers and in half of the Araçá stations. These values are high, even compared with those reported for domestic-sewage-contaminated marine or estuarine sediments, as observed, for example: in Bengal Basin, India (700–1000 μg g-1 [86]); in Mai Po marshes, Hong Kong (789–1737 μg g-1 [87]); and at Saco da Capela submarine outfall in São Sebastião Channel (Brazil) (334–926 μg g-1) [25]. The high TP concentrations recorded in the DTCS area are similar to those found in the highly polluted Guanabara Bay, Brazil (370–2300 μg g-1; [88]). According to Baturin [89], all of the stations, including the background point, have TP concentrations indicative of anthropogenic activities, i.e., higher than 700 μg g-1 (0.7%). It may result from the important input of phosphorus (both organic and inorganic fractions) discharged with produced waters. During petroleum exploration via offshore platforms, inhibitors enriched in phosphorus (phosphinopolycarboxylate) are used to prevent scale deposition (primarily BaSO4 and SrSO4) that can clog flow lines. Subsequently, the inhibitor aggregates in the crude oil can be discharged into the aquatic environment when the tanks are washed.

In all the stations except TB4, the inorganic fraction of phosphorus (IP) is higher than the organic fraction (OP). The dominance of IP has been associated in the literature with the disposal of untreated sewage containing detergents that include polyphosphates and orthophosphates [90], [91], [92]. The organic fraction, in turn, is primarily related to the biological processes of plant and animal tissues, which are related to increases in the concentrations of sewage, human waste, and food [90] [91]. These data suggest a chronic contribution of anthropogenic origin, mainly owing to the vicinity of the DTCS.

Considering that terrestrial OM is depleted in nitrogen, while marine zooplankton and phytoplankton have high nitrogen content [93], C/N (TOC/TN) values below 6 indicate marine OM and those above 15 indicate OM of continental origin. Intermediate values, between 7 and 14, indicate a mixture of both marine and continental origin. The values obtained for SSC indicate OM predominantly of marine origin while there is a mixture of OM sources around DTCS, and mostly a continental origin around Araçá outfall.

According to Ruttenberg and Goñi [94], sediments with marine phytoplankton contributions have a mean molar C/OP (TOC/OP) ratio of 106. In contrast, soft tissues from terrestrial sources are relatively impoverished in P, with characteristic mean molar C/OP values ranging from 300 to 1300. Woody terrestrials present values higher than 1300. Microbial communities that may be an important component of sedimentary OM have C/OP values ranging from 7 to 80. As a result, low C/OP levels may indicate anaerobic degradation of domestic sewage effluent [25]. Based on these works, and on the information given by the C/N ratio, it appears that the relatively low values of C/OP found in the study area probably result from a significant microbial contribution around both outfalls.

The C/S (TOC/TS) ratio provides information about redox conditions, especially regarding the importance of biological sulfate reduction in carbon decomposition, within sediments and in the overlying water column [53]. In general, aerobic marine sediments have C/S values greater than 5, whereas marine sediments undergoing sulfate reduction under euxinic/inhospitable bottom conditions (e.g., anoxic bottom waters with high H2S concentrations) have C/S ratios lower than 1.5 [95]. Some researchers have recorded C/S values ranging from 1.5 to 5.0, with an average value of 2.8 ± 0.8 in marine sediments undergoing sulfate reduction below an oxygenated water column [52], [95]. In the SSC, the Araçá region, and the area surrounding DTCS, C/S values ranging from 0.84 to 19.4 were recorded, indicating sediments deposited under oxygen-rich marine conditions (stations SSC1, SSC4–SSC8, SSC9, AR2, AR8, AR9, and TB5) or submitted to periodic anoxic marine conditions (stations SSC2, SSC9, AR1, AR3–AR6, AR10, TB1–TB4, and TB6–TB10). For the reference station, the C/S result of 6.6 indicates oxic conditions in the sediment and the water column. These results corroborate the observations based on Eh values recorded in the sediments of São Sebastião Channel and the DTCS area.

#### Trace elements

Except for Sr where they provide significantly different information, both index EF and Igeo are consistent in indicating that trace elements are moderately enriched in some stations, but may be mostly of natural origin. Only a few stations around Araçá outfall and DTCS diffusers are moderately enriched in As, Ba, Cd, Cu, Hg and Sr. The most enriched stations are AR6, AR8, TB4 and TB9. The moderate enrichment detected by EF and Igeo is supported by the fact that the trace element concentrations never exceed the PELs, and therefore never exceeded the upper limits set by CONAMA [58]. At 11 stations only, the As, Cu, and Ni concentrations were between their TELs and PELs. They were between the limits set by CONAMA [58] at only five stations. The toxicity of these elements to estuarine and marine organisms above certain thresholds [96] depends on their mobility, i.e., bioavailability [97], which is mainly influenced by the pH and potential redox (Eh) values of the environment [98].

Petroleum contains several metals, including Cr, As, Cd, Hg, and Cu. Chrome is associated with the matrix of crude oil; whereas As, Cd, and Cu are usually derived from contamination during production operations [99]. Most As and Cu result from corrosion inhibitors used in drilling operations or from biocides used in oil tankers [100]. The As content may range from 2.4 to 1,630 μg kg -1 (with an average of 15 μg kg –1) [101]. It is thus possible to infer that the As concentrations over TELs found at the study area results from the effluents of the DTCS. Crude oil and unprocessed gas condensates can contain significant amounts of suspended mercury compounds, primarily in the form of mercuric sulfide [102]. Thus, it is possible to infer that Hg enrichment found in the area may be directly related to the effluents from the DTCS.

Barium, despite having weak enrichment gradients as indicated by EF and Igeo, was present in relatively high concentrations, as compared to the reference station (SSC3), at several stations. The highest concentration was at station SSC10, positioned near the area where vessels dock before entering the channel. In this area, oil contamination was observed and was associated with the release of illegal ballast water from the washing tanks of oil tankers [103]. This contamination may be responsible of the higher Ba concentration, but motor oils from São Sebastião Harbor may also contribute to Ba enrichment. Concentrations of SO42-, Ba2+, and Sr2+ are generally high in produced waters [104], forming scale deposition of BaSO4 and SrSO4 that can clog flow lines and produce deposits in the bottom of petroleum tanks. Because wastewater from the DTCS partly originates from production water and water resulting from the washing of tanks of the terminal and of tankers, it may be a considerable source of these elements in the study area.

### Response of living benthic foraminifera to pollution impacts

The upper discussion shows that, despite potentially considerable pollution sources, mostly around DTCS, the contamination of sediment, as measured through geochemical analyses, is moderate. This probably results from the dispersion of effluents by the currents that affect the São Sebastian Channel. However, even if they are dispersed and do not accumulate within the sediment, pollutant may affect the benthos since all habitats exposed to all types of contaminants experience decreased biodiversity [105]. Indeed, the low densities of foraminifera around the DTCS diffusers, with an average of 9 ± 6 individuals per 10 cm3 of sediment, together with the low species richness and the low diversity and equitability indices, illustrate the impact of environmental stress on the benthos. Around the DTCS diffusers, 30 to 50% reduction of species richness and the dominance of a few species, leading to a decrease in equitability, were observed. Comparatively, foraminiferal parameters (i.e., density, species richness, diversity, and equitability) at the DTCS were lower than those observed in neighboring areas. Near the Araçá outfall, which discharges 104 L s-1 of domestic sewage [84], an average density of 62 ± 22 foraminifera per 10 cm3 of sediment was found, which was approximately sixfold higher than that at the DTCS. This suggests that the impact of the chemical pollution at the DTCS is stronger than the impact of sewage discharge at the Araçá outfall, even if this impact is noticeable when compared with SSC.

Organic matter rejected by sewage outfalls may either favor meiofauna [106], [107], or be responsible for lowering meiofauna density and richness [18], [21] [32]. A toxic threshold exists that depends on the concentration and nature of the organic matter present in the sediments [18], [108], [109]. Moreover, the degradation of organic matter requires large quantities of oxygen and, when the flux of organic matter exceeds the degradation rate, benthic hypoxia or even anoxia can occur. Due to toxicity and hypoxic condition, stenobiotic species of the meiofauna can disappear, leading to low species richness and diversity [12], [13], [18], [21], [32], [34], [35].

The impact of pollution is illustrated on the CCA biplot (Figs 8 and 9). The first ordination axis indicates the transition from slightly polluted, well-oxygenated sandy sediments (positively correlated with C/S), rich in Sr, with negative values, to reducing, muddy sediments, enriched in Cr, with positive values. The arrows representing the variables are long, and their angle to the axis is small, which indicates the significant impact of these variables on the environmental gradient. Positive values of axis 1 are clearly related to the pollution around the DTCS diffusers as it appears on Fig 8. The second ordination axis indicate the transition between both impacted areas, enriched in phosphorus (Araçá and DTCS), towards negative values, and the São Sebastião Channel, towards positive values.

The species negatively correlated with axis 1, i.e., *C*. *lobatulus*, *D*. *terquemi*, *R*. *floridensis*, *P*. *cananeiaensis*, and *H*. *boueana*, mostly epiphytic, clearly show their sensitivity to the pollution produced by the DTCS effluents. *Cibicides lobatulus*, which is the most strongly negatively correlated with axis 1 is known to be one of the most sensitive species to pollution by trace elements in the northwestern Adriatic Sea [26], [110], in the Ria de Aveiro, north of Portugal [111], and in the polluted harbor of Ile d´Yeu, France [112]. *Pararotalia cananeiaensis* is an herbivorous species that is characteristic of marine environments [73] [112]. It is abundant in dead assemblages all over the SSC. The weak positive correlation of *P*. *cananeiaensis* as well as *R*. *floridensis* with TP and Cd is an indication that they are tolerant to these elements and their associated contaminants derived from organic pollution. It is consistent with previous observations near the Araçá domestic submarine outfall, where *P*. *cananeiaensis* lived preferentially at stations with high contents of sulfur (*r* = 0.86; *p* <0.01), OP (*r* = 0.62; *p* <0.01), and silt (*r* = 0.75; *p* <0.01) [25]. However, the ecological preferences of *P*. *cananeiaensis* are still not well known.

The species correlated with positive values of axis 1 of the CCA, corresponding to reducing muddy sediment, with moderate concentrations of Cr and noticeable OM content, show only weak correlation. It shows that these species are more tolerant to pollution than other species (their relative abundance increases correlatively to the decreasing abundance of sensitive species), but does not indicate a preference for polluted areas. Species having the highest correlation with axis 1 are *B*. *marginata*, *B*. *elegantissima*, *B*. *compacta*, *A*. *tepida*, and *A*. *parkinsoniana*. Most of these species are recognized in the literature as being tolerant to high OM flux and as being able to survive in low oxic conditions [34], [35]. Bandy et al. [113, 114] noted that *B*. *elegantissima* and *B*. *marginata* tend to be abundant in areas affected by pollutants. *Ammonia tepida* is an eurybiotic species that is characteristic of nearshore areas and paralic environments [115]. The tolerance of *A*. *tepida* to adverse conditions, including organic and chemical pollution, has long been reported in both field studies and culture studies (e.g., [16], [18], [28], [107], [116], [117]). Its potential application for pollution monitoring is well established. Its plot position, not far from the center of the plane, towards the positive values of axis 1 and negative values of axis 2, which is mostly associated with DTCS samples, corresponds to its higher proportion in the polluted samples from DTCS and, to a lesser extent, from Araçá. The highest proportion of *A*. *tepida* and the decline of stenobiotic species indicate that both organic (Araçá) and chemical (DTCS) pollution significantly impacted the benthos.

The correlation of *P*. *cananeiaensis* with the negative values of axis 2 as already been discusses. Conversely, four species are strongly correlated with the positive values of this axis, i.e. negatively correlated with high concentrations of TP or Cd: *N*. *opima*, *H*. *pacifica*, *H*. *boueana* and *G*. *crassa*. They show a high sensitivity to the increases of TP and/or Cd concentrations and confirm the impact of the pollution on meibenthos in the study area.

## Conclusions

Despite the high pollution potential of the Araçá and DTCS effluents, geochemical analyses show that the sediments are only moderately impacted. Sediments near the DTCS were enriched with As, Cu, and Ni, these elements reaching concentrations higher than their TELS, but none of the measured elements reached their PELs. Concentrations of TOC, TN, and TP (especially phosphorus and sulfur) are atypically high in the sediments around the DTCS compared to those of SSC, Araçá, and the reference station, indicating nutrient enrichment. However, the enrichment is weak when compared with other areas in the world subjected to organic pollution.

We can thus conclude that 1) the enrichment exists, showing that wastewater treatment is not effective in removing some chemical elements from petrochemical liquid waste; 2) the impact on sediments shown by geochemical analyses is weak, probably dure to the dispersion of pollutants by the strong currents that affect the São Sebastião channel.

The finding of a weak impact on the geochemical characteristics of the sediment should lead to conclude that the Araçá and DTCS effluents do not significantly affect the environment. However, foraminiferal assemblages clearly show that the meiobenthos is affected, and consequently all the trophic web may be impacted.

## Supporting information

S1 Appendix Geographical coordinates and hydrological data of the sampled stations. (XLSX)

S2 Appendix Geographical coordinates of the sampled stations, sedimentological data: Textural, physicochemical parameters and geochemical results. (XLSX)

S3 Appendix Relative abundance (%) of the identified living benthic foraminifera and biotic parameters in the three studied regions. (XLSX)
